# Effects of Tumour Necrosis Factor Antagonists on Insulin Sensitivity/Resistance in Rheumatoid Arthritis: A Systematic Review and Meta-Analysis

**DOI:** 10.1371/journal.pone.0128889

**Published:** 2015-06-25

**Authors:** Agata N. Burska, Rajalingham Sakthiswary, Naveed Sattar

**Affiliations:** 1 Leeds Institute of Rheumatic and Musculoskeletal Medicine, University of Leeds, Leeds, United Kingdom; 2 Department of Medicine, University Kebangsaan Malaysia, Kuala Lumpur, Malaysia; 3 Institute of Cardiovascular and Medical Sciences, University of Glasgow, Glasgow, United Kingdom; VU University Medical Center, NETHERLANDS

## Abstract

**Objective:**

Beyond the joints, TNFi (tumour necrosis factor inhibitor) therapy may confer systemic benefits in rheumatoid arthritis (RA). Several studies have investigated the role of TNFi on insulin resistance/sensitivity (IR/IS). This question is of general interest given the emerging evidence linking inflammation and insulin resistance. The main aim of this review was to summarise the published data and to determine the effects of TNFi on IR/IS.

**Methods:**

We searched the PubMed and ISI Web of Knowledge databases for studies which examined the effects of TNFi on IR/IS. The studies were assessed independently by two reviewers according to a pre-specified protocol. The data on Homeostatic Model Assessment for Insulin resistance (HOMA) and Quantitative Insulin Sensitivity Check Index (QUICKI) were pooled and reported as standard difference in means (SDM) with 95% confidence interval (CI) using a random-effects model.

**Results:**

A total of eight studies with 260 subjects met the selection criteria. The duration of the studies was from 8 weeks to 12 months. There was statistically significant reduction in HOMA index in six out of eight studies and four reported significant increment in QUICKI. The pooled analysis revealed significant reduction in HOMA [SDM-0.148, 95%CI[-0.278 to -0.017], p=0.026] and increment in QUICKI [SDM 0.312, 95%CI[0.019 to 0.606], p=0.037] with TNFi.

**Conclusion:**

There is emerging evidence to support that TNFi therapy improves IS and reduces IR in RA. Further, well conducted trials are needed to determine if such effects translate to lower incidence of diabetes in RA or other autoimmune conditions on biologic therapy.

## Introduction

There is an established link between systemic inflammation and insulin resistance (IR). Several studies have highlighted an inverse relationship between disease activity scores in Rheumatoid Arthritis (RA) and beta cell function [1]. Severe insulin resistance is present even in early untreated RA patients [2]. Accumulating evidence supports a significant association between IR and the culprit cytokines in RA such as tumour necrosis factor (TNF) α, interleukin 1 and interleukin 6 [3–6]. The precise mechanism remains elusive but a complex adipokine-mediated interaction among adipose tissue, IR and RA has been implicated [7]. In RA, adipocytes and the surrounding macrophages produce a wide range of adipokines including resistin. Resistin interferes with glucose homeostasis by opposing the actions of insulin leading to IR [8].

TNF inhibitors (TNFi) have revolutionised the therapeutic arena of RA in the recent decades. Beyond the joints, TNFi therapy likely confers systemic benefits [9]. Several studies have examined the effects of TNFi therapy on IR. These studies were conducted using commercially available TNFis namely, infliximab, etanercept and adalimumab. Theoretically, TNF blockade enhances insulin sensitivity (IS) by increasing the tyrosine kinase activity of the insulin receptor and promotes insulin-glucose-mediated uptake in the skeletal muscle [10]. Improved insulin resistance, however, may not be solely due to the effects of TNFi. Effective suppression of the inflammatory process could be partially explanatory. Anti-TNF therapy may improve insulin resistance in RA patients by reversing defects in the phosphorylation/activation status of the insulin signaling pathway [11]. The vast majority of the studies in the literature assessed IR by the HOMA (Homeostasis Model Assessment of Insulin Resistance) and the QUICKI (Quantitative Insulin Sensitivity Check Index). The HOMA was developed from physiological studies into mathematical equations describing glucose regulation by estimating insulin resistance and β-cell function from fasting glucose and insulin levels [12]. The QUICKI, on the other hand, measures IS using the inverse of the sum of the logarithms of the fasting insulin and fasting glucose [13]. A recent systematic review shows that HOMA-IR and QUICKI have reasonable associations with clamp a ‘gold standard’ measure of peripheral insulin sensitivity [14].

The main objective of this systematic review is to evaluate the published data to determine the effects of TNFi on IR/IS. This is an interesting question since diabetes rates might be altered by anti-inflammatory therapy, in particular biologics, but randomised trials are sparse. We therefore sought to capture best available evidence on effects of biologics on the key pathways leading to diabetes, namely insulin resistance.

## Methods

### Data Sources and Searches

We followed the MOOSE (Meta-analysis of observational studies in epidemiology) guidelines for reporting systematic reviews and meta-analyses. Two authors (RS and ANB) independently screened articles for inclusion in this systematic review by going through the abstracts retrieved from the search strategy. The PubMed, ScienceDirect and ISI Web of Knowledge databases were searched for articles published from 1999 to 2014 designed as: clinical trials, randomized controlled studies, observational (prospective and cross-sectional) studies that evaluated the effects of TNFi on IR/IS in RA. Search terms included various combinations of the following: “TNF antagonists”, “adalimumab (ADA)”, “etanercept (ETN)”, “infliximab (IFX)”,”certolizumab”, “golimumab”, “insulin resistance (IR)”, “insulin sensitivity (IS)” and “rheumatoid arthritis (RA)”. Articles identified during the initial screening process as potentially relevant were brought forward for full-text review. Disagreements among the authors were resolved by consensus. The bibliographies of relevant studies and narrative reviews were searched for further relevant publications.

### Study selection

The following studies were eligible for inclusion:

1. studies involving RA patients.
2. longitudinal studies which investigated the association between TNFi therapy and either the HOMA index and/or the QUICKI.
3. studies published in English.

**Fig 1:**
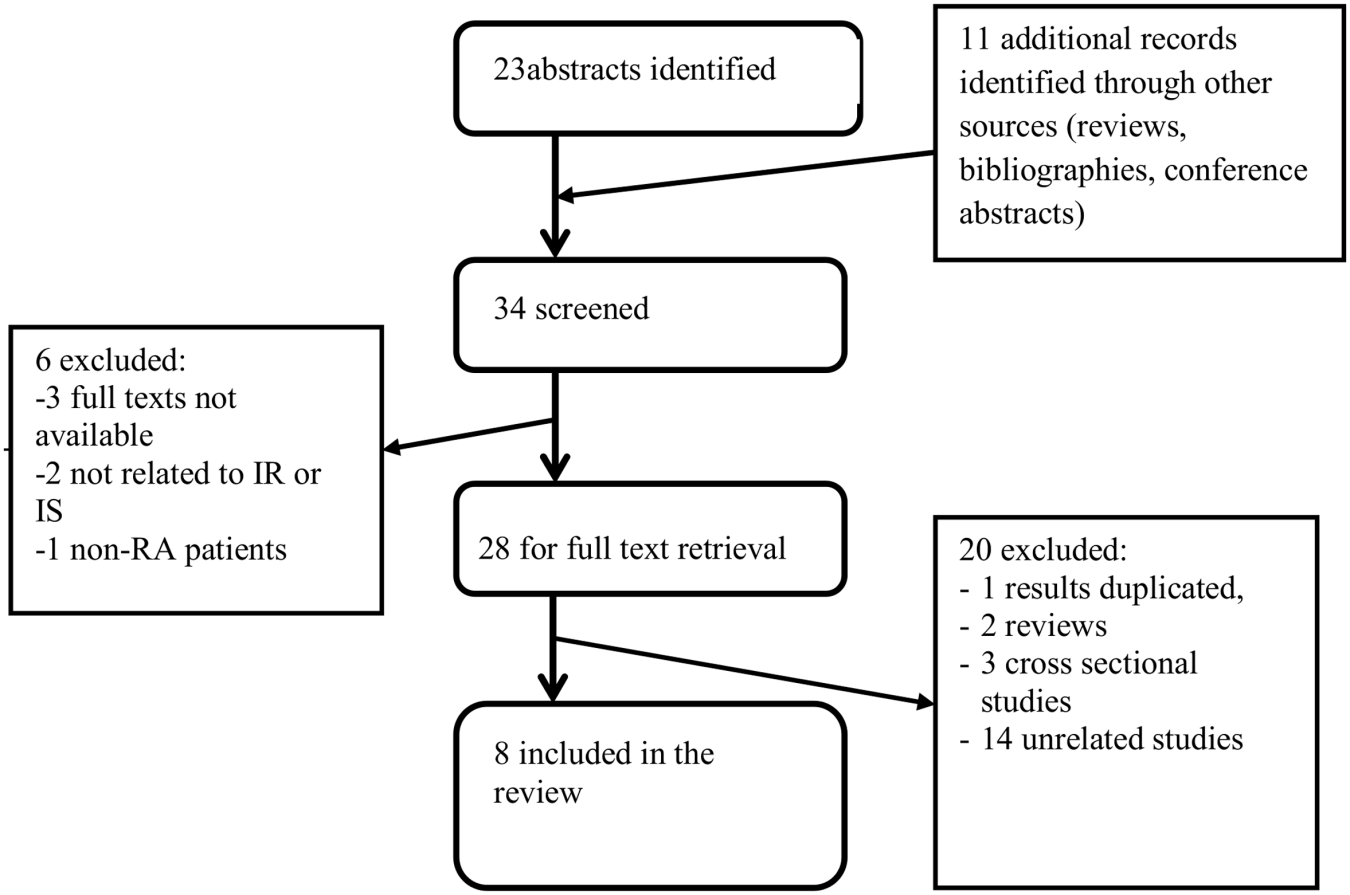
Flow chart of studies identification and selection.

We excluded conference abstracts, case reports, cross-sectional studies and proceedings. Search strategy is presented on Fig 1.

### Data extraction and Quality Assessment

We extracted the following data from the selected studies: year, study design, study population, sample size, type of TNF antagonist, comparator drug, and the study duration. The following outcome measures were used to evaluate the effect of TNF inhibition on IR/IS; the HOMA and QUICKI indices. The values of these indices as well as the *p* values were collected. As for studies which provided the median values, we estimated the means following the formula by Hozo et al [15]. The quality of the controlled studies was assessed based on the “Instrument to measure the likelihood of bias”, proposed by Jadad *et al* [16].

### Data Synthesis and Analysis

**Fig 2:**
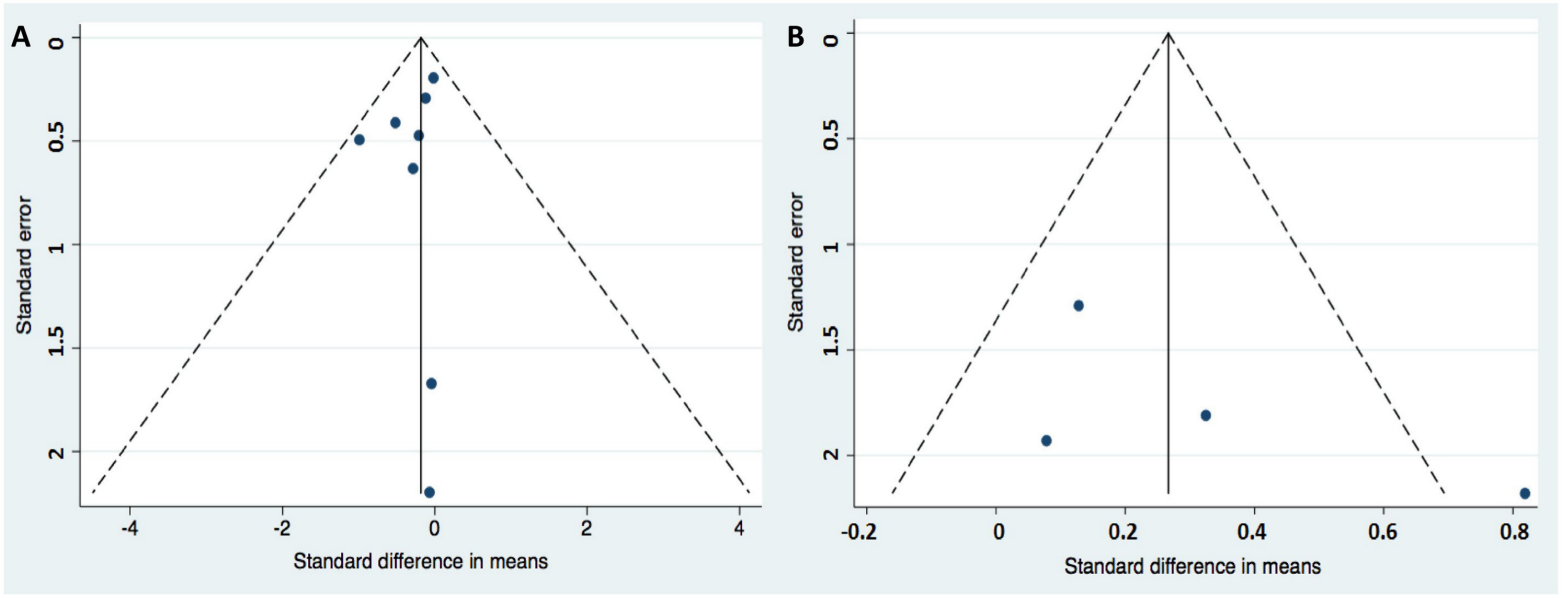
Funnel plots (A) for HOMA from 8 selected studies and (B) for QUICKI from 4 selected studies evaluating effects of TNFi on IR/IS.

Data were pooled using a random effects model for a more conservative but precise estimate of the effects of TNF antagonist on IR/IS. This model allows for heterogeneity across the studies[17]. The data on the outcome of TNF antagonist therapy versus controls were expressed as standard difference in means (SDM) with 95% confidence intervals (CIs). Comprehensive Meta-analysis software version 2.0 was used to analyse the data and generate the Forest plots of the pooled data. Egger’s test was used to evaluate publication bias for the meta-analysis of the HOMA and QUICKI indices Fig 2).

## Results

Based on the prespecified eligibility criteria, a total of eight studies were selected for this systematic review [11, 18–24]. All these studies are presented in Table 1. Four of the eight studies [11, 19, 21, 24] employed control groups. The controls were patients on conventional DMARDs i.e. methotrexate, leflunomide, sulfasalazine, healthy controls or on non-TNFi biologic i.e. abatacept. All the controlled trials were neither randomised nor double blinded and did not describe the withdrawals and drop-outs. Hence, the Jadad score was 0 for these studies [11, 19, 21, 24]. The TNFis which were investigated in this series include etanercept (four studies with 34 subjects) [11, 18, 19, 24], infliximab (seven studies with 147 subjects) [11, 18–20, 22–24] and adalimumab (four studies with 29 subjects) [11, 18, 19, 21]. Despite certolizumab pergol and golimumab being included in our search, we were not able to identify a single study evaluating insulin sensitivity/resistance with these pharmaceuticals. Across the studies, the sample size ranged from seven to 61 subjects. The duration of the studies was from 8 weeks to 12 months. The study by Seriolo et al was not included in the statistical analysis as no standard deviation (SD) values were provided in the paper.

**Table 1:** Summary of the studies evaluating the Effects of TNFi Therapy on IR/IS in RA.

				HOMA-IR			QUICKI		
Author, year and ref	Number of subjects	Concomitant medications in the TNFi arm	Duration of follow up	Pre TNFi	Post TNFi	*p* value	Pre TNFi	Post TNFi	*p* value
				Mean (SD)			Mean (SD)		
Stavropoulos-Kalinglou et al. 2012 [18]	IFX n = 20	NA	6 months	2.6 (0.60)	2.4 (0.80)	0.088	0.33 (0.02)	0.36 (0.02)	0.092
	ETN n = 11								
	ADA n = 1								
				Mean (95%CI)			Mean (95%CI)		
Stagakis et al. 2012 [11]	IFX n = 49	NA	12 weeks	7.00 (5.20–9.40)	-5.68 (-8.86- [-2.50])	**0.001**	0.29 (0.28–0.30)	0.07 (0.04–0.09)	**0.001**
	ADA n = 11								
	ETN n = 1								
	Controls ABA n = 7								
				Median (IQR)					
Ferraz-Amaro et al. 2011 [19]	ADA n = 8	NA	12 months	1.60 (0.80)	1.60 (0.60)	0.24	---		
	IFX n = 6								
	ETN n = 2								
	Controls Biologic Naive n = 34								
	Healthy subjects n = 70								
				Mean			Mean		
Seriolo et al. 2008 [24]	ETN n = 20	Stable doses of NSAID and MTX (10 mg/wk)	24 weeks	1.44	1.73	**0.01**	0.36	0.37	**0.01**
	IFX n = 18								
	Controls TNFi Naïve n = 20								
				Mean (95%CI)					
Rosenvinge et al. 2007 [21]	ADA n = 9		8 weeks	1.40 (0.30)	1.30 (0.30)	0.05	---		
	Controls Healthy Subjects n = 9								
				Mean (SD)					
Oguz et al. 2007 [22]	IFX n = 7		9.6 months (mean)	2.40 (1.00)	1.10 (0.50)	0.05	---		
				Median (IQR)					
Tam et al. 2007 [20]	IFX n = 19	All subjects on the same dose of NSAID and oral prednisolone ≤10 mg/day.	14 weeks	1.34 (0.63–2.13)	0.58 (0.43–1.30)	0.05	---		
				Mean (SE)			Mean (SE)		
Kiortsis et al. 2005 [23]	IFX n = 28	Prednisolone (5 mg/day) and CSA or MTX	6 months	3.01 (0.48)	1.89 (0.35)	**0.01**	0.30 (0.01)	0.35 (0.01)	**<0.010**

ABA-Abatacept, ADA-Adalimumab, CI-Confidence Interval, CSA-Cyclosporine, ETN-Etanercept, HOMA-Homeostatic Model Assessment for insulin resistance, IFX-Infliximab, IQR-interquartile range, IR-Insulin Resistance, IS-Insulin Sensitivity, MTX-Methotrexate, NSAID-Non Steroid Anti-Inflammatory Drugs, QUICKI-Quantitative Insulin Sensitivity check Index, SD-Standard Deviation, SE–Standard Error, TNFi-Tumour Necrosis Factor α inhibitor.

Five out of eight of the studies [20, 21, 24–26]allowed low and stable doses of prednisolone of less than 10 mg throughout the studies. In the study by Ferraz-Amaro et al [27], none of the subjects received steroids. Two of the remaining studies (Stavropoulos-Kalinglou et al and Stagakis et al)[11, 18] did not provide any information on the use of steroids. Six out of 8 [11, 18, 21, 25, 26, 28] of the included studies excluded subjects with diabetes mellitus.

### Effects of TNFIs on HOMA

**Fig 3:**
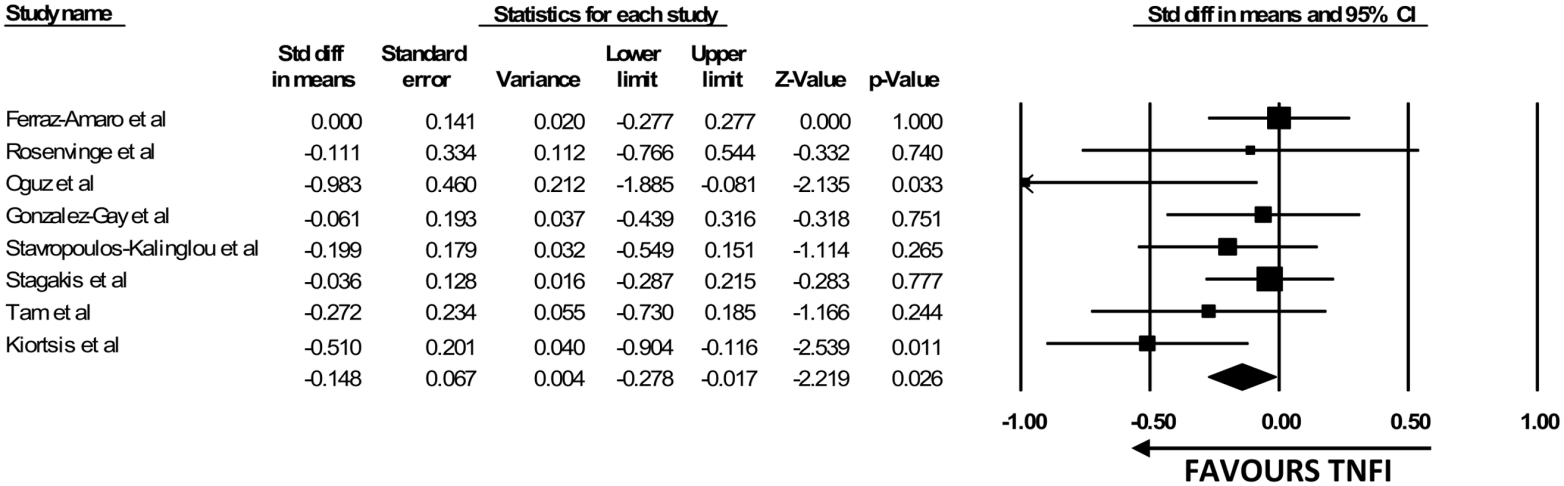
The Forest Plot for the effect of TNFi on HOMA index.

All 8 studies assessed insulin resistance by HOMA index with a combined total of 210 subjects [11, 18–24]. Out of the 8 studies, 6 showed significant decrease in HOMA index after TNFi treatment [11, 18, 20, 22–24]. The mean duration of the studies was 24.05 weeks (~6 months). A significant change in the HOMA index was detected as early as 8 weeks. Our pooled analysis with 7 studies [11, 18–23] revealed a significant improvement in HOMA index with TNFi therapy (SDM -0.191, 95%CI [-0.370 to -0.012], p = 0.037) (Fig 3). The inter-study heterogeneity test was not statistically significant with a *p* value of 0.255 and I^2^ of 21.98%.

### Effects of TNFIs on QUICKI

**Fig 4:**
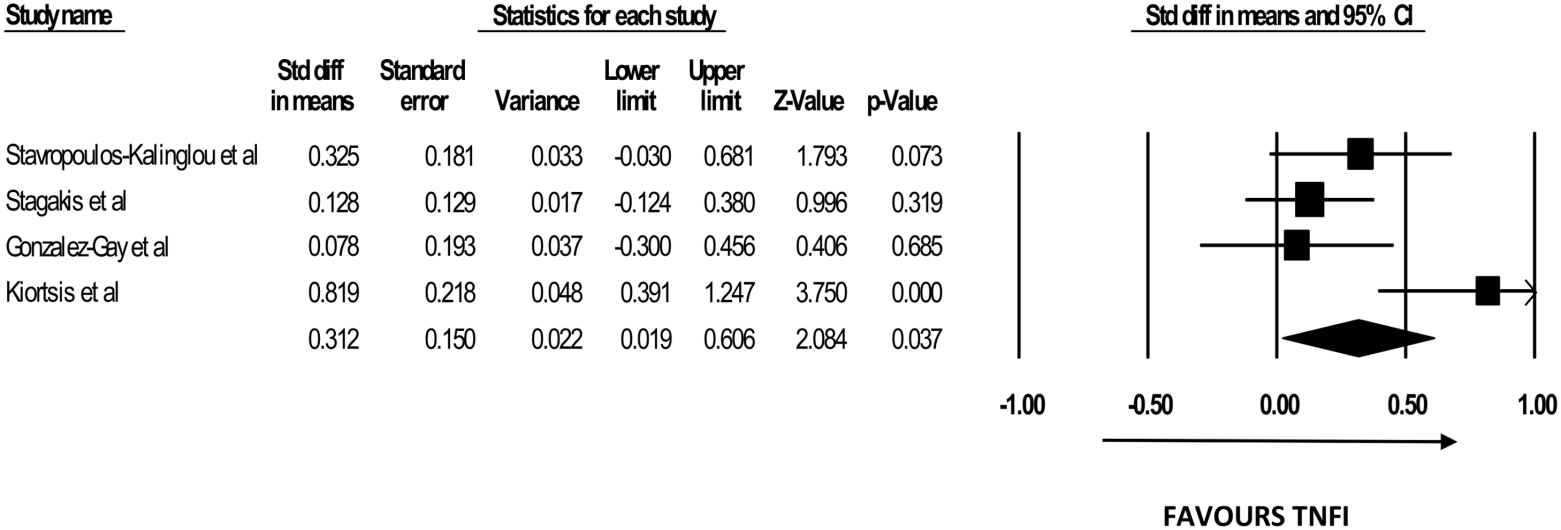
The Forest Plot for the effect of TNFi on QUICKI.

Only 4 studies [11, 18, 23, 24] used QUICKI to evaluate insulin sensitivity. QUICKI was calculable for a total of 159 subjects. Significant increase in QUICKI values was recorded in all 4 [11, 18, 23, 24, 29] of the studies. The mean duration was 21 weeks (5.25 months). The study with the shortest duration showing significant changes in QUICKI lasted for 12 weeks [11]. Our pooled analysis with 3 studies [11, 18, 23] showed a significant increase in QUICKI with TNFi (SDM 0.393, 95%CI [0.014 to 0.772], p = 0.042) Fig 4). The heterogeneity test yielded a value of I^2^ = 65.19% which was statistically significant (p = 0.035).

## Discussion

This systematic review and meta-analysis highlighted that there is evidence supporting the beneficial effects of TNFi therapy on insulin sensitivity in RA. The pooled analysis of both the outcome measures i.e. HOMA and QUICKI were consistent, though heterogeneity in QUICKI was evident. The HOMA index which reflects insulin resistance, decreased significantly in all except for 2 studies. Nevertheless, the combined results suggest an improvement in insulin sensitivity with TNFi treatment. The relevance of these studies is that impaired glucose tolerance is more prevalent among patients with RA compared to the healthy controls [30]. Studies have consistently demonstrated that the degree of the impaired glucose metabolism was related to the severity of the inflammatory activity [31, 32]. It is therefore predicted that biologics would lessen such abnormalities in patients with RA. Our work also support the findings of a potentially beneficial effect of TNFi on the risk of diabetes mellitus (lower by 51%) based on data from an inception RA cohort [33].

The reduction in the HOMA, however, may not be exclusively seen only with TNFi therapy in RA. Cross sectional studies by Perez et al and Cuchacovich et al [34, 35] found no difference in the HOMA index between the TNFi arm and the methotrexate (MTX) treated arm. In a recent study by Rajappa et al [36] systemic MTX therapy was associated with a significant reduction in insulin levels and insulin resistance indices in patients with psoriasis vulgaris. Whether the same holds true in RA remains unknown owing to the lack of well-designed randomised controlled trials in this regard. It is noteworthy that Seriolo et al [24], who performed a head-to-head comparison between TNFi and MTX therapy, demonstrated a significant decrease in the HOMA index compared to baseline at 24 weeks in the TNFi arm (1.445 vs 1.733; p<0.01) but there were no changes observed in the controls on MTX therapy. This finding however, should be interpreted cautiously given that the control arm was on a relatively low dose of MTX i.e. 10 mg weekly.

Across the studies which investigated the changes in QUICKI with TNFi, there was broad consistency. All 5 studies reported a significant increment in this parameter. The improvement in insulin sensitivity with TNFi therapy observed in RA does not echo the findings in other disease conditions such as diabetes mellitus and inflammatory bowel disease [37–39]. Ofei et al. [37] reported that the fasting glucose, fasting insulin and C-peptide levels were unaffected by TNFi therapy in obese Type 2 diabetics. Along this line, Stavropoulos-Kalinglou et al. [18] reported that improvement in HOMA and QUICKI were seen only in the normal weight IR patients and not in the obese subjects.

Gentile et al [38], on the other hand found that infliximab did not interfere with insulin secretion, insulin resistance and production of antibodies to glutamic acid decarboxylase and islet cells in patients with Crohn's disease. It is tempting to speculate that TNFi therapy was ineffective in enhancing insulin sensitivity in the above conditions due to impaired insulin signalling through other mechanisms independent of TNFα or systemic inflammation.

We do acknowledge the limitations of this systematic review. Studies included in this review were mainly observational. Observational studies are prone to methodological bias and cannot prove causality. Further the study designs were rather heterogenous making direct comparisons difficult. In addition, most of the studies did not perform adjustments for potential confounding factors in particular changes in body mass index, smoking, or by capturing cumulative dose of TNFi, and/or changes in corticosteroids. The results of this systematic review should be interpreted with great caution as the aforementioned confounders were not adjusted for in the analyses. Therefore, the present findings should be seen as hypothesis generating and data from randomised trials are needed to definitively prove a link between inflammation suppression via biologics and changes in insulin resistance.

## Conclusion

This systematic review provides further evidence that TNFi therapy likely enhances IS in patients with RA. The work support the need for further trials in this area and to link such changes to clinical end-points, in particular risk for diabetes in patients with RA and other auto-immune conditions.

## Supporting Information

S1 Appendix Excluded articles. (DOCX)

S2 Appendix PRISMA checklist. (DOC)
