# Supplementary material for: Effects of Tumour Necrosis Factor Antagonists on Insulin Sensitivity/Resistance in Rheumatoid Arthritis: A Systematic Review and Meta-Analysis
Source: PLoS One. 2015 Jun 25;10(6):e0128889. doi: 10.1371/journal.pone.0128889 (PMC4482317; doi:10.1371/journal.pone.0128889)
Supplement: S1 Appendix — (DOCX) [file pone.0128889.s001.docx]

**Appendix S1 : Excluded Articles**

**Cross sectional studies**

1. Gonzalez-Gay MA, Gonzalez-Juanatey C, Vazquez-Rodriguez TR, Miranda-Filloy JA, Llorca J. Insulin resistance in rheumatoid arthritis: the impact of the anti-TNF-alpha therapy. Ann N Y Acad Sci.2010 Apr;1193:153-9.

2. Gonzalez-Gay MA, Gonzalez-Juanatey C, Miranda-Filloy JA, Llorca J. The potential effect of TNF-alpha antagonist therapy in rheumatoid arthritis may depend on the degree and severity of insulin resistance before the onset of this therapy. Horm Metab Res.2012 Jun;44(7):558-9

3. Gonzalez-Gay MA, De Matias JM, Gonzalez-Juanatey C, Garcia-Porrua C, Sanchez-Andrade A, Martin J, et al. Anti-tumor necrosis factor-alpha blockade improves insulin resistance in patients with rheumatoid arthritis. Clin Exp Rheumatol.2006 Jan-Feb;24(1):83-6.

**Review articles**

4. Channual J, Wu JJ, Dann FJ. Effects of tumor necrosis factor-alpha blockade on metabolic syndrome components in psoriasis and psoriatic arthritis and additional lessons learned from rheumatoid arthritis. Dermatol Ther.2009 Jan-Feb;22(1):61-73.

5. Hollan I, Meroni PL, Ahearn JM, Cohen Tervaert JW, Curran S, Goodyear CS, et al. Cardiovascular disease in autoimmune rheumatic diseases. Autoimmun Rev.2013 Aug;12(10):1004-15.

**Abstracts without full text.**

6. Aggarwal BB. Tumour necrosis factors receptor associated signalling molecules and their role in activation of apoptosis, JNK and NF-kappaB. Ann Rheum Dis.2000 Nov;59 Suppl 1:i6-16.

7. Matsumoto I, Zhang H, Yasukochi T, Iwanami K, Tanaka Y, Inoue A, et al. Therapeutic effects of antibodies to tumor necrosis factor-alpha, interleukin-6 and cytotoxic T-lymphocyte antigen 4 immunoglobulin in mice with glucose-6-phosphate isomerase induced arthritis. Arthritis Res Ther.2008;10(3):R66.

8. Inoue A, Matsumoto I, Tanaka Y, Iwanami K, Kanamori A, Ochiai N, et al. Tumor necrosis factor alpha-induced adipose-related protein expression in experimental arthritis and in rheumatoid arthritis. Arthritis Res Ther.2009;11(4):R118.

**Duplication of results**

9. Seriolo B, Paolino S, Ferrone C, Cutolo M. Effects of etanercept or infliximab treatment on lipid profile and insulin resistance in patients with refractory rheumatoid arthritis. Clin Rheumatol.2007 Oct;26(10):1799-800.

**Unrelated studies**

10. Neidel J, Blum WF, Schaeffer HJ, Schulze M, Schonau E, Lindschau J, et al. Elevated levels of insulin-like growth factor (IGF) binding protein-3 in rheumatoid arthritis synovial fluid inhibit stimulation by IGF-I of articular chondrocyte proteoglycan synthesis. Rheumatol Int.1997;17(1):29-37.

11. Daza L, Martin-Jimenez R, De la Torre PX, Hernandez E, Murillo B. Improvement of ACTH response to insulin tolerance test in female patients with rheumatoid arthritis due to tumor necrosis factor inhibition. Eur J Endocrinol.2007 Jul;157(1):47-51.

12. Wang SW, Lin TM, Wang CH, Liu HH, Houng JY. Increased toll-like receptor 2 expression in peptidoglycan-treated blood monocytes is associated with insulin resistance in patients with nondiabetic rheumatoid arthritis. Mediators Inflamm.2012;2012:690525.

13. Marcora SM, Chester KR, Mittal G, Lemmey AB, Maddison PJ. Randomized phase 2 trial of anti-tumor necrosis factor therapy for cachexia in patients with early rheumatoid arthritis. Am J Clin Nutr.2006 Dec;84(6):1463-72.

14. Sarzi-Puttini P, Atzeni F, Scholmerich J, Cutolo M, Straub RH. Anti-TNF antibody treatment improves glucocorticoid induced insulin-like growth factor 1 (IGF1) resistance without influencing myoglobin and IGF1 binding proteins 1 and 3. Ann Rheum Dis.2006 Mar;65(3):301-5.

15. Maruotti N, d'Onofrio F, Cantatore FP. Metabolic syndrome and chronic arthritis: effects of anti-TNF-alpha therapy. Clin Exp Med.2014 Nov 27.

16. Famenini S, Sako EY, Wu JJ. Effect of treating psoriasis on cardiovascular co-morbidities: focus on TNF inhibitors. Am J Clin Dermatol.2014 Feb;15(1):45-50.

17. Peters MJ, van Sijl AM, Voskuyl AE, Sattar N, Smulders YM, Nurmohamed MT. The effects of tumor necrosis factor inhibitors on cardiovascular risk in rheumatoid arthritis. Curr Pharm Des.2012;18(11):1502-11.

18. Solomon DH, Massarotti E, Garg R, Liu J, Canning C, Schneeweiss S. Association between disease-modifying antirheumatic drugs and diabetes risk in patients with rheumatoid arthritis and psoriasis. Jama.2011 Jun 22;305(24):2525-31.

19. Vassiliou EK, Gonzalez A, Garcia C, Tadros JH, Chakraborty G, Toney JH. Oleic acid and peanut oil high in oleic acid reverse the inhibitory effect of insulin production of the inflammatory cytokine TNF-alpha both in vitro and in vivo systems. Lipids Health Dis.2009;8:25.

20. Cuchacovich R, Espinoza LR. Does TNF-alpha blockade play any role in cardiovascular risk among rheumatoid arthritis (RA) patients? Clin Rheumatol.2009 Oct;28(10):1217-20.(unrelated)

21. Spanakis E, Sidiropoulos P, Papadakis J, Ganotakis E, Katsikas G, Karvounaris S, et al. Modest but sustained increase of serum high density lipoprotein cholesterol levels in patients with inflammatory arthritides treated with infliximab. J Rheumatol.2006 Dec;33(12):2440-6.

22. Shiojiri T, Wada K, Nakajima A, Katayama K, Shibuya A, Kudo C, et al. PPAR gamma ligands inhibit nitrotyrosine formation and inflammatory mediator expressions in adjuvant-induced rheumatoid arthritis mice. Eur J Pharmacol.2002 Jul 19;448(2-3):231-8.

23. Suzuki S, Morimoto S, Fujishiro M, Kawasaki M, Hayakawa K, Miyashita T, et al. Inhibition of the insulin-like growth factor system is a potential therapy for rheumatoid arthritis. Autoimmunity.2014 Oct 29:1-8.

24. Papagoras C, Voulgari PV, Drosos AA. Atherosclerosis and cardiovascular disease in the spondyloarthritides, particularly ankylosing spondylitis and psoriatic arthritis. Clin Exp Rheumatol.2013 Jul-Aug;31(4):612-20.

25. Boyer JF, Cantagrel A, Constantin A. Impact of traditional therapies and biologics on cardiovascular diseases in rheumatoid arthritis. Curr Vasc Pharmacol.2008 Jul;6(3):218-27.

26. Genre F, Lopez-Mejias R, Rueda-Gotor J, Miranda-Filloy JA, Ubilla B, Villar-Bonet A, et al. IGF-1 and ADMA levels are inversely correlated in nondiabetic ankylosing spondylitis patients undergoing anti-TNF-alpha therapy. Biomed Res Int.2014;2014:671061.
